# Intra-Abdominal Adhesions

**Published:** 1921-01-15

**Authors:** 


### A FACTOR IN SURGICAL FAILURES.

### Intra-Abdominal Adhesions.

The dual purpose of this note is to explain to the health worker some not well appreciated facts in connection with abdominal operations, and to remind the less specialised surgeon of some of the more elusive points of his art. There are two circumstances which make it remarkable that the formation of that well-known pre- or post-operative complication generally known as "intra-abdominal adhesions" does not possess a more voluminous literature. The first arises with the fact that in almost all pathological processes involving intra-abdominal structures and attended by inflammatory reactions, immediate or subsequent adhesions between neighbouring layers of peritoneum may be expected to occur. The second is that their formation is liable to develop after, and complicate, the result of almost any intra-abdominal operation, and their prevention is therefore of no little interest to the patients concerned.

Expert opinion is generally agreed upon the view that attempts, automatically, to shut off inflammatory *foci* by adhesions represent a simple and natural defensive action in animal economy, and that all such adhesions are essentially benign in their intent. In the operating-theatre their action is clearly illustrated by processes commonly noted around a chronically inflamed appendix or ovary, and it certainly appears that failure on the part of Nature thus to localise the infecting micro-organisms allows of septic invasion of the system, frequently with a fatal ending to the illness. But, for the benefit of the general reader, it is well to describe in outline the process by which an adhesion arises.

Essentially it is a definite organisation of connective and scar tissue lying between and joining up the surfaces of two or more adjacent viscera. The first step in its formation arises with exudation of serum from the adjacent membranous surfaces; this as the result of inflammation or direct injury. The serum coagulates, filling up the natural space between the viscera, and meanwhile into it escape a variety of blood and connective tissue cells, which ultimately form a definitely organised tissue, bridging the gap which was normally present. In the end a strong "adhesion" of fibrous and connective tissue remains.

### The Essential Causes.

Letting this outline description suffice for the moment, the next important question occurs with the various factors which may indirectly produce or augment the exudation. As previously explained, even mild or local bacterial infect is extraordinarily potent, and another reason for observing *absolute* asepsis in abdominal surgery is therefore apparent. For the same reason there is little doubt that, through some degree of bacterial invasion, the formation of adhesions is promoted by the employment in the finally closed wound of either capillary or tubal drainage tubes; and this practice might well be entirely dispensed with, except where the most urgent and positive necessity exists. Also, direct trauma to the surfaces has been mentioned, and it is to be remembered that a degree of injury caused merely by excessive handling of the viscera during operation is sufficient to produce exudation of serum, and, later, adhesive formation. But these are all causes which arise directly at the time of operation, and it must not be forgotten that in the untreated person, repeated inflammatory attacks in the abdominal organs may produce any number of adhesions, as may also contusions resulting from blows or wounds in the abdomen. Obviously, this matting together of surfaces, crowding of or kinking of vital structures, can cause a variety of symptoms which, however, it is beyond the scope of this note to describe, and we had best briefly review the preventive methods at the surgeon's command, apart from general observance of asepsis, gentleness, and care.

### Attempts at Prevention.

At various times surgical fashion has allowed many extraneous substances to be run into the abdominal cavity with a view to preventing adhesions. Lane's introduction of sterile normal saline is a simple example, but results did not fulfil the expectations. Substances, such as various oils, paraffin, lanolin, gum arabic, etc., have earned a host of critics of their use, and many consider that they more often augment than prevent the trouble. Sodium citrate has been claimed as inhibiting fibrin formation and postponing the production of scar tissue, but at the same time its use makes the control of hæmorrhage particularly difficult. In 1914 there was some talk of using amniotic membrane (a thin fœtal envelope) to cover the surfaces of viscera denuded of peritoneum, but little more has been heard of the method.

### Definite Experiments.

More definite on these points is some recent experimental work from America in connection with operations upon animals. The surgical technique employed was in strict accordance with that demanded for human subjects. In the first or control experiment a very careful end-to-end intestinal anastomosis was performed, and an autopsy six weeks later showed the abdomen to be free from adhesions, the intestinal surfaces normal, and all peritonitis absent. A second experiment was performed in which the omentum (a natural fold of peritoneum normally passing down in front of the intestines) was fixed so to cover over the operation area. In other cases free grafts of this omentum were fixed over the disturbed intestinal surfaces, and in none were adhesions found at a later *post-mortem* examination.

In contrast with the above, similar experimental operations were performed using injections of sterile

**A Factor in Surgical Failures**—(*continued*).

paraffin, olive oil, etc., but in almost every case adhesions were noted, some of the animals even dying of peritonitis. Similarly, the use of sodium citrate was found to favour rather than prevent the matting together of injured visceral surfaces, and from their work the authors more or less justifiably concluded that among the best, if not the only methods by which adhesions may be prevented are (*a*) the limiting of wounds in the peritoneum by careful technique during the operation; and (*b*) by covering over such wounds as are necessarily caused by a normal peritoneal surface such as the omentum or a graft from the same membrane.

This much, then, appears simple, and at least it finally disposes of the claims of sundry preventive methods, but, nevertheless, the whole subject of post-operative adhesions still retains many puzzling contradictions. Sometimes they may follow most trivial procedures, and yet in other instances they may not be found after operations needing quite forcible manipulation of the abdominal contents. It must be admitted that there is still much to be discovered before they can with any certainty be avoided.

### The General Survey.

At present, however, the broad lines already indicated must be adherred to, and in the attempt to do so it is worth while to remember the following points, which are small and, therefore, often overlooked. Viscera should not be exposed to the air, and its drying action, a moment longer than necessary. Bleeding, even from the minutest points, should be most carefully stopped before the final wound closure, for blood-clots during absorption will cause the densest of adhesions. Only moist sponges and pads should be used, for dry gauze is intensely irritating to a peritoneal surface. Every raw surface should have peritoneum most carefully stitched over it. Viscera, particularly intestines, should not be allowed to come into contact with irritating antiseptics, the iodine on the wound edges is a frequent indirect cause of trouble in this way. Also there is the necessity early after operation to re-establish a continuance of peristalsis, or involuntary movements in the intestines themselves. The patient's position in bed should be changed slightly but frequently, and his general posture chosen as the one most favourable to avoidance of crowding together of the viscera in an operation area.

Many more such small points might be raised, each applicable to some special type of case; but, on the whole, there appears little doubt that excellence in surgical technique, and care and common sense in wound closure and after-treatment, at present form our best means to ward off a disappointing complication in an otherwise successful case. Here these matters have not been dealt with from the practised surgeon's point of view, but rather in a superficial manner, where attention is drawn to the essentials of the situation and the relative futility of certain injections at operation of extraneous substances into the abdominal cavity. Let the surgeon concentrate on his actual surgery, and meticulous care will be most times rewarded.

